# The effect of opium dependency on testis volume: a case-control study

**Published:** 2012-11

**Authors:** Ali Cyrus, Hassan Solhi, Mahdi Azizabadi Farahani, Hamid Reza Khoddami Vishteh, Davoud Goudarzi, Ghasem Mosayebi, Hamed Mohamadian

**Affiliations:** 1 *Department of Urology, School of Medicine, Arak University of Medical Sciences, Arak, Iran.*; 2 *Department of Toxicology and Forensic Medicine, School of Medicine, Arak University of Medical Sciences, Arak, Iran.*; 3 *Arak University of Medical Sciences, Arak.Iran.*; 4 *Department of Microbiology and Immunology, Arak University of Medical Sciences, Arak, Iran*; 5 *Kermanshah University of Medical Sciences, Kermanshah, Iran.*

**Keywords:** *Testis volume*, *Male reproductive system*, *Opium*, *Cigarette*

## Abstract

**Background:** Given the paucity of data on possible testis changes in opioid dependents, we sought to compare the testis volumes between a group of opium dependents and a group of healthy controls.

**Objective:** Comparison of testis volume between opium dependents and healthy controls.

**Materials and Methods:** This case-control study recruited 100 men with opium dependency (cases) and 100 healthy men (controls) in Iran, in 2008. A checklist containing questions about age, height, weight, daily amount of cigarette use, and duration of cigarette use for all the participants as well as daily amount of opium use (grams) and duration of opium use (years) for the case group was completed. Additionally, the dimensions of each testis were measured by a single person using calipers, and the mean of the left and right testes volume was compared between these two groups.

**Results:** The mean of the testis volumes in the case group was significantly lower than that of the case group (11.2±2.2 and 25.1±2.7cm³, p<0.001). The results of the ANCOVA test showed that even after the omission of the cigarette smoking effect (p=0.454), the testis volume remained lower in the opium dependents (R^2^=0.884, p<0.001). In the case group, there were significant reverse correlations between testis volume and age (r=-0.404, p<0.001), daily amount of opium use (r=-0/207, p=0.039) and duration of opium use (r=-0.421, p<0.001).

**Conclusion:** We found that the testis volume in the male opium dependents was lower than that of the healthy controls. We would recommend that future studies into the impact of drugs on the testis dimensions pay heed to possible histological changes in the testes owing to opium dependency.

## Introduction

According to the World Drug Report in 2007 an approximate 200 million people in the world (5% of the world’s total population) aged 15 and above had used substances in the year before (1). A US social study found that more than 4.3 million people used opioids continuously although many of them were on drugs prescribed by physicians or non-specialists such as family and friends (2, 3). 

The American Attitudes on Substance Abuse XIII underscored the availability of prescription opioids for abuse as reported by teens and parents alike (4). Indeed, the teens in the study claimed that prescribed drugs were more accessible than beer. 

What is more, maintenance treatment by opioids for opioid dependency was once reported to have accounted for a minimum of 460,000 cases in Europe and 241,000 cases in the USA (5, 6). Undesirable side effects of opioids intensify concerns about the high prevalence of opioid use. 

A case in point is endocrine gland dysfunction and its concomitant sexual dysfunction, which has been the subject of much research (7). About two thirds of patients under opioid treatment were once reported to suffer from sexual dysfunction, with the dysfunction signs lingering after treatment in one third of them (8).

An estimated 5 million people in the US and Canada are believed to be inflicted with this dysfunction (9, 10). The bulk of the research conducted on the effects of opioids on the male reproductive system has focused on the impact on the hypothalamus and pituitary gland or, in other words, the central nervous system, which controls the male productive system (11-13). 

To the best of our knowledge, there is a dearth of data in the existing literature on changes in the testes, as one of the major endocrine glands, in opioid dependents and there is also a lack of comparative studies into possible differences in the testis volume between opioid dependents and control groups (14, 15). The present study was, therefore, carried out to compare the testis volumes between a group of opium dependents and a group of healthy controls. 

## Materials and methods

**Design and setting**

This case-control study was conducted by opioid dependency treatment clinics in the Iranian city of Arak in 2008. Informed written consent was obtained from all the participants, and the study protocol was approved by ethical committee of Arak University of Medical Sciences.

**Participants**

There were 100 men in each study group. The case group was comprised of opium dependents selected from opioid dependency treatment clinics in Arak, and the control group comprised male inpatients without a history of any drug use in orthopedic wards of Vali-Asr Hospital of Arak. 

The inclusion criteria for the case group were in accordance with the DSM-IV criteria for opioid dependency, with the minimum fulfillment requirement being three of the following conditions within the previous year: withdrawal, unsuccessful withdrawal attempts, opioid use more than or longer than the prescribed amount or duration by a physician, lengthy period of recovery from the side effects of opioids, and social or occupational or recreational dysfunction (16). The exclusion criterion for both study groups was a history of the diseases that affect the testis volume. As a result, a history of any of the following was to be ruled out: varicocele, inguinal hernia, inguinal region or testis surgery, orchitis, mumps orchitis, undescended testis, liver cirrhosis or liver failure, clinical signs of hypogonadism (such as absence of pubic and axilla hair), and gynecomastia. 

**Measurements and outcomes**

The research assistants introduced themselves and fully explained the aims to the participants before completing a checklist of basic characteristics such as age, height, and weight in addition to variables pertaining to cigarette use, for instance daily amount and duration of use. Information on opium use such as daily amount (grams) and duration of use (years) was also recorded for the case group. The main outcome was the mean of the volumes of the right and left testes as measured by a single examiner in both groups. The examiner measured the testes by tensioning the scrotum skin completely by his left hand and measuring the dimensions of each testis with calipers (17). The mean volume of the right and left testes was measured according to the length and width of each testis (18). 

**Statistical analysis**

Statistical analysis was conducted using SPSS for Windows 13 software. The quantitative variables were described in range and mean (SD). The independent t-test was utilized to make comparisons between the two groups with respect to age, body mass index, cigarette use, and testis volume. The ANCOVA  test was employed to assess the independent effect of cigarette use by considering the case and control groups as fixed factors, cigarette use as a simultaneous variable, and testis volume as a dependent variable. Furthermore, the bivariate correlation test helped assess the association between testis volume and independent quantitative variables in the case group. P-value<0.05 was considered significant.

## Results

**Basic characteristics**

The age ranges of the case and control groups were 20-49 and 21-48 years, respectively. The respective body mass index ranges of the case and control group were 16.8-31.1 and 18.3-29.3 kg/m². The ranges of the amount of cigarette use were 0-5 and 0-8 packs/year  in  the case and control groups, respectively. The range and mean (SD) of the amount of opium use in the case group were 1-12 and 2.7±1.8 gr/day, and the range and mean (SD) of the duration of opium use were 1-28 and 8.7±6.2 years. No significant difference was seen between the case and control groups in terms of age and body mass index (p<0.001) (Table I).

**Dimensions and volumes of testes:**

The mean (SD) of the right and left testis volumes in all participants were 18.2±7.4 and 18.1±7.4cm³, respectively. The volumes of the right (p<0.001) and left (p<0.001) testes as well as the mean of the volumes of both testes (p<0.001) in the case group were significantly lower than those of the control group (Table II).

**Independent effect of cigarettes on testis volume**

The results of the ANCOVA test for an assessment of the simultaneous effect of cigarette and opium use on the testis volume revealed that the testis volume in the patients with opium dependency was significantly lower than that of the control group (R²=0.884, p<0.001), despite the deletion of the effect of cigarette use (p=0.454).

**Factors relating to testis volume in opium dependency**

In the opium dependents, there was a significant reverse correlation between age (r=-0.404, p<0.001), daily amount of opium use (r=-0.207, p=0.039), and duration of opium use (r=-0.421, p<0.001) and testis volume. The values of body mass index (p=0.501) and amount of cigarette use (p=0.501) showed no significant relation with the testis volume.

**Table I:** Comparison of basic characteristics between opium dependents and healthy controls

	**Opium dependents (n=100)** **Mean (SD)**	**Healthy controls (n=100)** **Mean (SD)**	**p-value** *
Age (year)	32.9 (7.3)	33.0 (5.3)	0.929
Body mass index (kg/m²)	22.7 (3.1)	23.3 (2.6)	0.135
Cigarette use (pack/ year)	14.8 (13.7)	0.9 (1.7)	<0.001
Right testis volume (cm³)	11.3 (2.3)	25.1 (2.7)	<0.001
Left testis volume (cm³)	11.2 (2.3)	25.0 (2.9)	<0.001
Mean testis volumes (cm³)	11.2 (2.2)	25.1 (2.7)	<0.001

* Independent T-Test.

**Table II:** Comparison of testis dimensions and volumes between opium dependents and healthy controls

		**Opium dependents (n=100)**		**Healthy controls (n=100)**		**p-value** *
		**Range**	**Mean (SD)**	**Range**	**Mean (SD)**	
Right testis						
	Height (cm)	3.8-4.8	4.1 (0.2)	4.7-5.4	5.1 (0.2)	<0.001
	Width (cm)	2.0-2.8	2.3 (0.2)	2.7-3.4	3.1 (0.1)	<0.001
	Volume (cm³)	8.0-18.5	11.3 (2.3)	18.3-32.7	25.1 (2.7)	<0.001
Left testis						
	Height (cm)	3.7-4.7	4.1 (0.2)	4.8-5.4	5.1 (0.1)	<0.001
	Width (cm)	2.0-3.0	2.3 (0.2)	2.7-3.4	3.1 (0.2)	<0.001
	Volume (cm³)	8.0-21.2	11.2 (2.3)	18.3-32.1	25.0 (2.9)	<0.001
	Mean testis volumes (cm³)	8.0-19.8	11.2 (2.2)	18.3-32.4	25.1 (2.7)	<0.001

* Independent T-test.

## Discussion

The testis volume in the opium dependent group was significantly lower than that of the healthy controls in our study. Cigarette use was more common among our case group, but an assessment of the independent effect of cigarette and opium use on the testis volume revealed that not only did the amount of cigarette use did not affect the testis volume but also after the omission of the effect of cigarette use, opium use still exerted a significant effect on the testis volume. 

Furthermore, an evaluation of the basic characteristics and variables pertaining to cigarette and opium use showed that although increasing age and amount and duration of opium use could lead to a reduced testis volume, body mass index and cigarette use had no impact on the testis volume. There is currently no disagreement in the scientific community about the negative impact of all opioids on the function of endocrine glands and sexual health (19, 20).

The testis, an endocrine gland secreting sexual hormones and producing sperms, is certainly no exception. For all the evidence in the existing literature on the reduction in the volume and morphological changes in the testes in opioid dependents (14, 15, 21). Our literature review showed that there was a lack of comparisons in this regard between opioid dependents and healthy controls and hard evidence had yet to be presented of reduction in the testis size. It is deserving of note, however, that a likely culprit in opioid dependents is testis dysfunction (22). 

Opioids, whether produced endogenously or exogenously, are capable of banding to opioid receptors in the hypothalamus, pituitary, and even testis and influence the function of the sexual glands (23). A decrease in sexual hormone levels or interference in the pulsatory secretion of GnRH hormone at the hypothalamus level and the resultant reduction in the secretion of FSH and LH hormones from the pituitary gland are some known effects of opioids on the reproduction system. 

Moreover, these substances can directly affect the testis tissue and lessen the production and secretion of sexual hormones as well as the interstitial fluid of testes (14). Previous research has already shed light on such acute and chronic effects of opioids on the endocrine system as reduced FSH and LH levels in the wake of a drop in GnRH levels and also their direct impact on the testis in animals (24, 25). 

Research also shows that an increase in the duration of opium use precedes a decrease in sexual hormone levels (26, 27). Much controversy still exists about the effect of cigarette consumption on the normal reproduction system and testes. While some investigators maintain that smoking exerts a significant negative impact on different parts of the reproductive system others have shown not only a normal function of the reproductive system but also no difference in the size of testes in cigarette smokers in comparison with non-smokers (28). As regards animals, however, there is a consensus about the pernicious effects of nicotine and cigarette smoke on the reproduction system such as testicular atrophy (29).

To the best of our knowledge, the present study is the first of its kind to assess the testis volume in opioid dependents with a sufficient sample volume. Be that as it may, it suffers from some limitations such as non-measurement of the values of different hormones secreted by the endocrine glands (e.g. FSH, LH, and testosterone) and absence of testis-function assessor tests (e.g. sperm analysis) in opioid dependents. 

## Conclusion

The findings of this study showed that the testis volume in the men with opium dependency was significantly lower than that of those without opium dependency. In addition, whereas an increase in age and amount and duration of opium use was in tandem with a continuous reduction in the testis volume in the opium dependents, body mass index and amount and duration of cigarette use had no effect on the testis dimensions. We would, therefore, recommend that people under treatment for problems created by opium during the withdrawal process receive special attention to testicular atrophy through consultation with an urologist alongside treatment for other mental and physical side effects.
